# Cable failure tolerant control and planning in a planar reconfigurable cable driven parallel robot

**DOI:** 10.3389/frobt.2023.1070627

**Published:** 2023-05-17

**Authors:** Adhiti Raman, Ian Walker, Venkat Krovi, Matthias Schmid

**Affiliations:** ^1^ Clemson University, Automotive Engineering, Greenville, SC, United States; ^2^ Clemson University, Electrical/Computer Engineering, Clemson, SC, United States

**Keywords:** Reconfigurable CDPR (Cable Driven Parallel Robot), IMM (interacting multiple model) algorithm, LQR (linear quadratic regulator) control, FTC (fault tolerant control), FDD (fault diagnosis and detection), EKF (extended kalman filter), redundancy resolution, switching control

## Abstract

The addition of geometric reconfigurability in a cable driven parallel robot (CDPR) introduces kinematic redundancies which can be exploited for manipulating structural and mechanical properties of the robot through redundancy resolution. In the event of a cable failure, a reconfigurable CDPR (rCDPR) can also realign its geometric arrangement to overcome the effects of cable failure and recover the original expected trajectory and complete the trajectory tracking task. In this paper we discuss a fault tolerant control (FTC) framework that relies on an Interactive Multiple Model (IMM) adaptive estimation filter for simultaneous fault detection and diagnosis (FDD) and task recovery. The redundancy resolution scheme for the kinematically redundant CDPR takes into account singularity avoidance, manipulability and wrench quality maximization during trajectory tracking. We further introduce a trajectory tracking methodology that enables the automatic task recovery algorithm to consistently return to the point of failure. This is particularly useful for applications where the planned trajectory is of greater importance than the goal positions, such as painting, welding or 3D printing applications. The proposed control framework is validated in simulation on a planar rCDPR with elastic cables and parameter uncertainties to introduce modeled and unmodeled dynamics in the system as it tracks a complete trajectory despite the occurrence of multiple cable failures. As cables fail one by one, the robot topology changes from an over-constrained to a fully constrained and then an under-constrained CDPR. The framework is applied with a constant-velocity kinematic feedforward controller which has the advantage of generating steady-state inputs despite dynamic oscillations during cable failures, as well as a Linear Quadratic Regulator (LQR) feedback controller to locally dampen these oscillations.

## 1 Introduction

Cable driven parallel robots (CDPRs) are lightweight mechanisms with favourable power-to-weight ratios. They can be designed and structured to be manipulable over large workspaces, which can prove useful in application domains such as painting, bricklaying, material hauling, warehousing and concrete 3D printing. Large workspace domains bring with them a set of challenges such as increased cable sag, unmodeled cable dynamics and the increased effects of environmental factors such as unmodeled vibrations and air resistance. Heavy and dynamically-changing payloads may also cause cables to experience high tensions while possibly exciting unmodeled dynamic effects, such as swinging payloads (if the system is under-constrained) or unexpected cable collisions with surrounding objects in the workspace. Here, the addition of geometric reconfigurability is able to offer the flexibility needed to overcome some of these challenges. Geometric reconfigurability in this context is the ability of a robot to change its configuration through kinematic redundancy which is introduced by actuating the cable attachment points on the base frame of the CDPR. This robot is known as a *reconfigurable CDPR* or *rCDPR* in this work. Although most CDPRs can be manually reconfigured to accommodate different workspace reachability requirements by changing their geometric attachment points (as designed in (Gagliardini et al., 2016)), automating this process for online reconfiguration provides unique advantages that are still only minimally explored. For example, the robot can not only move to new configurations or new locations in the workspace without manual intervention, but can also gain capabilities that would otherwise not be possible (Rasheed, 2019), such as changing the quality (or performance index) of the pose at the end-effector without effecting its position or orientation (Zhou et al., 2014; Raman et al., 2020). Online reconfigurability introduces the properties of kinematic redundancy (i.e. singularity avoidance, self-motion) to a naturally actuation redundant system (i.e. the kinematically controllable degrees-of-freedom at the end-effector of a CDPR is less than the number of cables that support it). In this work, we exploit geometric reconfigurability to introduce a new control strategy for fault tolerance against cable failures.

**FIGURE 1:**
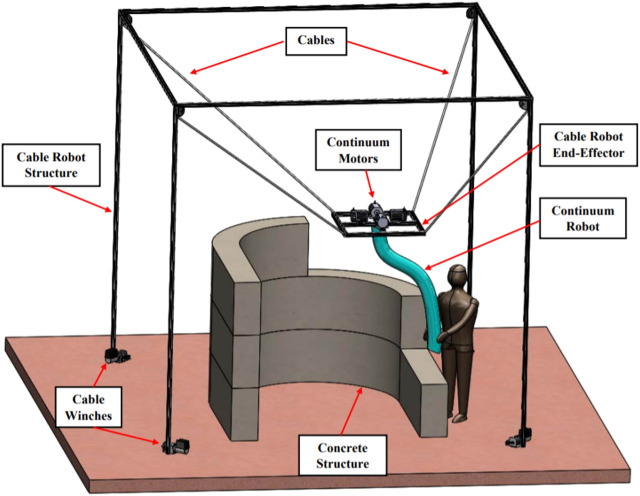
Micro + macro manipulator concept drawing for concrete 3D printing.

This research is motivated by the development of a cooperative robot (cobot) assisting in concrete delivery tasks such as concrete 3D printing or human-directed concrete application (Figure 1). The cobot is expected to consist of two subsystems: (i) a cable driven parallel manipulator that controls a payload over a large workspace (called the *macro*-manipulator); and (ii) a continuum robot enclosing a concrete delivery tube that provides finely directed control through congested spaces (called the *micro*-manipulator) (Srivastava et al., 2022).

**FIGURE 2:**
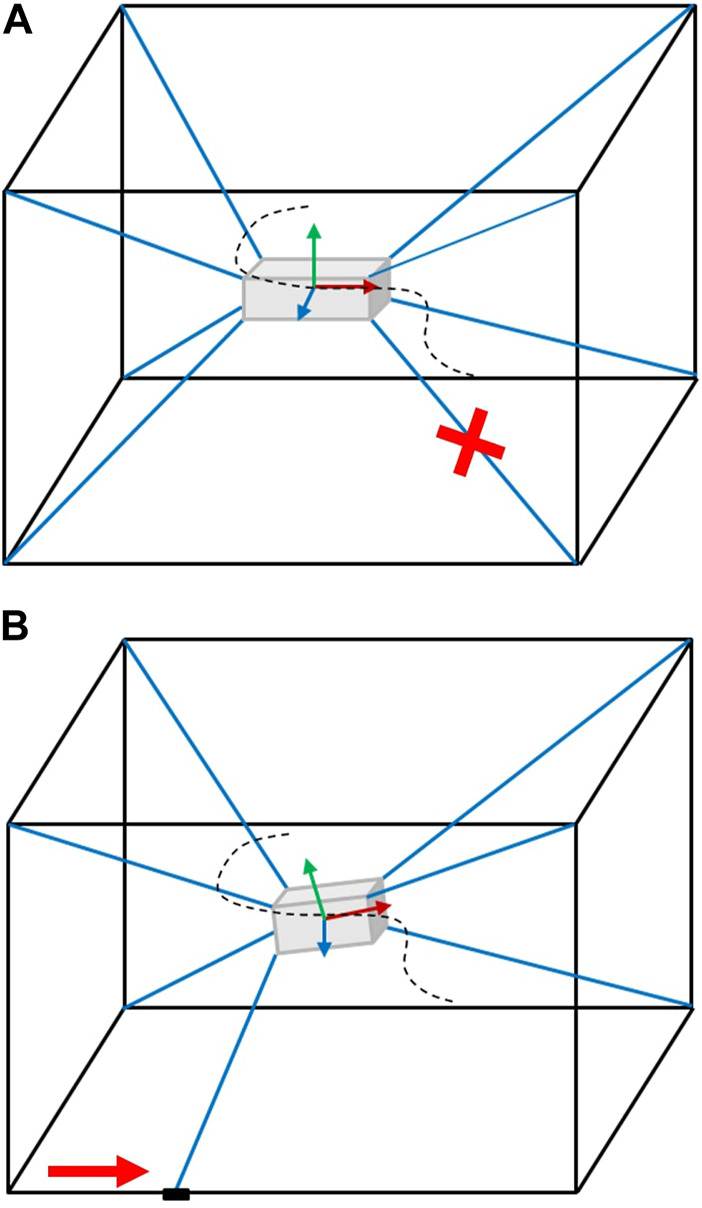
Geometric reconfigurability allows the trajectory tracking to continue. **(A)** A cable fails. **(B)** However, sliders reconfigure to allow the trajectory tracking to continue.

This work contributes to the development of the first subsystem by focusing on research questions involving the challenges posed by the addition of kinematic redundancies to conventional CDPRs (Raman et al., 2020; 2021; Walker et al., 2022). Online geometric reconfiguration can recover the static workspace and mobility lost through cable failures while also optimizing the quality of that mobility to a feasible extent. The basic idea is pictorially demonstrated in Figure 2. This form of fault tolerant control (FTC) may be unique to the addition of kinematic reconfigurability in CDPRs.

In our previous work (Raman et al., 2022) we demonstrated a proof-of-concept implementation of the automatic cable failure tolerant control framework that could simultaneously identify and overcome cable failures in an automatically without requiring an accessory diagnostic fault detection protocol. Failure identification, pose estimation, and cable failure tolerant control are accomplished simultaneously in this framework by relying on pose measurements from the end-effector alone. An interactive multiple model (IMM) adaptive estimation algorithm with a set of extended Kalman filters (EKFs), where each EKF corresponds to a different motion model, is applied to this measurement data and integrated into the control scheme which in turn provides input information from a set of controllers dependent on the different motion models of the failure modes.

In this research, we build upon that body of work by eliminating some design assumptions and increasing the uncertainties in model and noise measurements. The plant model is simulated to be as realistic as possible by incorporating disturbances, actuator dynamics and non-linearities arising from cable elasticity to demonstrate the real-world feasibility of the framework. In addition, we track complete trajectories despite cable failures by automatically returning to the point of failure to continue the task. The framework is updated to include two controllers (i) a feed-forward controller which has the advantage of input stability in the face of dynamic oscillations during cable failures and (ii) an LQR-based feedback controller stabilized around the desired joint inputs that is helpful in damping dynamic oscillations during a failure event and in recovering the trajectory quickly. The addition of the feedback controller eliminates the need for the resource-intensive feedforward kinematic controller to run at every timestep.

## 2 Related work

Fault tolerant control in robotics has a long history (Visinsky et al. (1994)). The use of kinematic redundancy to compensate for joint failures in serial robots is also well-established ***Maciejewski (1990). Early studies accounting for cable failures in CDPRs (Roberts et al. (1998)) showed that static equilibrium can be maintained (upon failure) if the robot is aligned to specific singular configurations. Bosscher and Ebert-Uphoff (2004) noted two kinds of failure modes: cable breakage due to excessive positive cable tensions or slackness due to the lack of cable tension. These failure modes could be precluded by limiting the desired wrenches at the end-effector to lie within the available wrench set Bouchard et al. (2010); Notash (2012) examined additional modes of cable failure such as stuck actuators (applying a passive restraint on the end-effector) or situations in which the actuator moves but the output is biased. However, these works are limited to kinematic models of CDPRs with ideal and inelastic cables. In a later work, Caro and Merlet (2020) outlined all possible dynamic failures for cable driven suspended robots and presented a practical passive solution to raise the end-effector load and ensure human safety in the event of cable failure. The authors noted the challenges of relying only on cable length measurements through the encoders or the loss of cable tensions to recognize that a cable failure has occurred. The authors instead recommended measuring the cable angles through IMUs affixed on the cables to detect a cable failure.

Outside of the context of cable failure, the challenges of relying on encoder and force measurements have been noted extensively in the context of CDPR control. Le Nguyen and Caverly (2021); Korayem et al. (2018); Caverly and Forbes (2016) addressed the futility of relying on forward kinematics from cable length measurements to determine end-effector pose, reasoning that encoder measurements do not necessarily correspond to the true cable length, which may be affected by unknown deformations and stiffnesses. To mitigate this issue, pose estimation techniques based on Extended (EKF) and Unscented (UKF) Kalman Filters applied on data from payload mounted IMUs were implemented. Korayem et al. (2018) further incorporated filtering with linearized feedback control for CDPRs, demonstrating that payload mounted IMUs can improve pose estimations in the presence of moderate-to-high dynamic effects.

Other literature on CDPR cable failures focused on dynamic failure recovery strategies or dynamic collision avoidance strategies. For instance, Passarini et al. (2019) studied emergency stop strategies for preventing collisions in cable suspended robots, whereas Boumann and Bruckmann (2022) focused on a force control strategy in the remaining cables of a cable-suspended robot to minimize the safety risk of dynamic effects during cable failures in the workspace. The authors assumed that the cable failures, when triggered, were always correctly identified and the proposed control framework was restricted to a post-failure setting.

Kinematic redundancies have also been shown to enable robust fault tolerant systems in parallel kinematic manipulators (PKMs). Notash (2011), for instance, investigated the effect of joint failures on the motion of kinematically redundant PKMs by analyzing the behavior of the changed kinematic model and setting task priorities to recover complete or partial motion. However, literature on the use of such redundancies for fault tolerant control (FTC) in cable driven manipulators is non-existent.

In this work we have explored a popular approach to failure detection and diagnosis (FDD) that has been utilized abundantly in aerospace applications. This FDD technique employs Multi Model Adaptive Estimation (MMAE) through the use of a bank of filters, with each filter correlated to a possible failure mode that may occur. In this study, we utilize an Interactive MMAE or IMM for short. Within robotics, IMMs have been employed for tracking trajectories (Mazor et al. (1998)) and behaviour prediction (Gill et al. (2019)) as well as in actuator fault detection and identification (Tudoroiu and Khorasani (2005)). Many of these studies focus on FDD only and do not attempt to integrate controllers for task recovery. One of the few exceptions is the work of Hill et al. (2021), where a non-linear Model Predictive Controller is applied to recover from reaction wheel failures that are identified through an UKF-based IMM for satellite maneuvering; an approach that is similar to our proposed strategy.

In this study, the cables are modeled as elastic springs. Furthermore, we assume that the employed dynamic models for control and estimation are imperfect and do not correspond perfectly to physical reality (e.g., through the inclusion of parametric uncertainties and process noise). We also assume that encoder readings do not correspond accurately to the true position of the end-effector and constitute an unreliable source of information; a reasonable assumption to make when accounting for elasticity in cables.

The inclusion of elastic behavior in the cables presents an additional control challenge. A feedforward controller that employs a simplified inelastic model does not account for this flexibility, causing the internal dynamics to present as undesired oscillations or vibrations at the end-effector. A feedback controller can be challenging to design when the local cable stiffness parameters (at time *t*_*k*_) are unknown or uncertain and the mapping from input to end-effector state is highly non-linear. The challenges of dynamic feedback control in CDPRs are well studied in literature, but their application has largely been focused on systems with cables of high stiffness. Feedback linearization Begey et al. (2018); Picard et al. (2020), sliding mode, and *H*_*∞*_ controllers have been successfully employed. Feedforward dynamic controllers like input-shaping have shown success (Baklouti et al. (2021)) in minimizing unwanted dynamics, especially in underactuated CDPRs (Idà et al. (2021)). In all these systems, the CDPR dynamics is often well known and the topology is unchanging making the system predictable and controllable. Nevertheless, model uncertainties and measurement noise can continue to present a challenge and can be overcome by the inclusion of an additional state estimation framework Kosari et al. (2013). Another approach to optimal control is the Linear-Quadratic-Gaussian (LQG) approach. In a recent work Chen et al. (2022) successfully employed an LQG with time-varying LQR gains on a CDPR and demonstrated improved performance compared to a baseline feedforward controller. Korayem et al. (2017) employed LQG alongside a feedback linearized controller to demonstrate improved tracking performance in the presence of model uncertainty and measurement noise. In this work we use LQRs for local stability by employing the state estimates from the IMM for state feedback. The steady-state control inputs that the LQRs are stabilized around are generated through kinematic feedforward controllers.

In this research, the applied IMM utilizes a parallel bank of Extended Kalman Filters, with each filter corresponding to one of the various cable failure modes. The mixed estimate from the IMM provides a corrected pose estimate together with an understanding of the current working mode. A parallel bank of feedforward controllers computes a set of possible joint inputs to the plant model (robot simulation) based on the desired trajectory information from the waypoint following algorithm. This forms the steady-state input that is modulated through a set of LQRs to generate updated inputs to minimize dynamic oscillations. The control inputs from the LQR are mixed with the IMM weights to control the robot. This approach simultaneously detects the fault, provides a corrected estimate and updates the input such that the system can robustly cope with sudden failures.

This framework is implemented in a simulation-only setting and thus serves as a proof-of-concept implementation of this framework. The highlights of this paper are as follows: (i) an Interactive Multi Model Filter is derived and demonstrated as a robust estimator for cable failure detection and diagnosis (FDD) as well as for real-time pose estimation of the rCDPR end-effector; (ii) a waypoint following and a constant-velocity path tracking algorithm is introduced to maintain constant velocity while path tracking and to recover the trajectory from the waypoint closest to the break-point after a cable failure event; (iii) a bank of kinematic feedforward controllers generate steady-state control inputs through kinematic redundancy resolution of each rCDPR model corresponding to each failure mode; and finally (iv) and LQR-based local stabilization framework is applied on the non-linear system to minimize local oscillations at the plant and hasten the recovery.

## 3 System models

**FIGURE 3:**
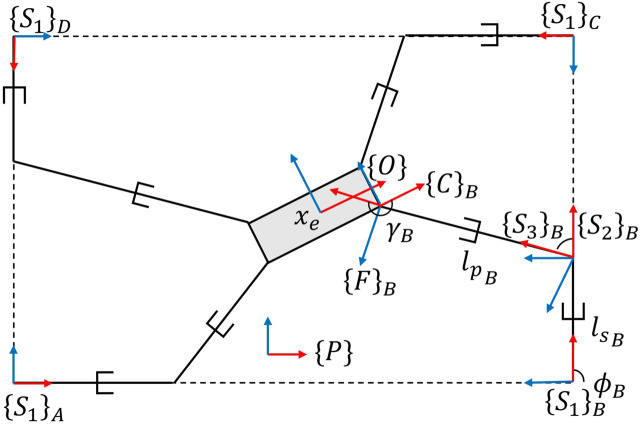
The kinematically redundant 4 cable CDPR.

This section introduces the mathematical model of the robot utilized in this research. The robot is a planar kinematically redundant CDPR with a platform that is driven by 4 cables and 4 linear actuators housing the cable winch mechanism. The system model is described in Figure 3. When the robot is fully functional, the system possesses four cables which are actuated by cable winches mounted on platforms on a linear actuator. The end-effector is a planar rigid body whose center is given by the origin of frame {*O*}. Of the three available degrees of freedom ({*x*, *y*, *ϕ*}), only two ({*x*, *y*}) are actively controlled. The cables are modeled as linear springs whose spring constant is a function of the current free (untensioned) cable length. The material constant of the cable stiffness is given by *k*_0_, where *k*_0_ is interpreted as the stiffness per unit reciprocal length of free cable. This indicates that longer cable lengths lead to lower stiffness in the cable and thus higher elastic deformations for the same tension. The current stiffness of the cable is therefore given by $k=\frac{k_{0}}{l_{p,0}}$ where *l*_*p*,0_ is the current free length of the cable. The true cable length *l*_*p*_ is the length of the prismatic joint from ${\left\{S_{3}\right\}}_{i}$ to {*F*}_*i*_ as seen in Figure 3 such that *l*_*p*_ = *l*_*p*,0_ + Δ*l*_*p*_ where Δ*l*_*p*_ is the cable elongation due to tension. Many approaches to modeling cable stiffness assume that the true cable length and free cable length are equal in the calculation of the current cable stiffness Verhoeven (2006), which helps decouple the dependence of cable stiffness on the cable tension and helps linearize the system. This assumption is not made here. The free cable length *l*_*p*,0_ was determined to be closer to the encoder value given by *l*_*c*_ = *rθ* where *r* is the winch radius and the interdependence of cable stiffness and cable tension is maintained. The tension in the cables is represented by *τ* = *k* (*l*_*p*_ − *l*_*c*_) where the condition *l*_*c*_ < *l*_*p*_ is maintained as much as possible to prevent cable sag. The cable tension is modulated by a unit step function, such that when this constraint is violated, the cable tension becomes zero, mimicking a realistic consequence of cable sag.

The cable-winch attachment points, {*S*_2_}, are not constant as in traditional CDPRs, but instead move along linear actuators along the *x*-axis of the base frames {*S*_1_}. The longitudinal positions of these attachment points with respect to the base frame are given by *l*_*s*_. Both the slider joints and the cable winching mechanisms are subject to actuation dynamics which inform the cable tensions and cable stiffnesses that effect the dynamics at the end-effector.

### 3.1 Actuator dynamics

Let the complete joint input vector from the controller (i.e. desired reference values to the plant model) be expressed as $q=\left[\begin{array}{cc} l_{s} & l_{c} \end{array}\right]$ and the realized joint vector (actual values at time *t*) be given by $q^{\prime }=\left[\begin{array}{cc} l_{s}^{\prime } & l_{c}^{\prime } \end{array}\right]$ . The moving slider bases are modeled as single degree-of-freedom (DOF) second order linear systems subject to maximum and minimum velocity constraints to mimic the motion of a screw-type linear actuator. The bases are modeled with inertia and friction and a simple proportional controller as shown in Eq. 1. For every *i*th actuator in the vector **l**_**s**_ (dropping the subscript *i* to indicate each individual slider joint) we model the dynamic equation as,

$$\begin{array}{ll} & m{\ddot{l}}_{s}^{\prime }+d{\dot{l}}_{s}^{\prime }=g_{p,ls}\left(l_{s}-l_{s}^{\prime }\right)\\ & \text{s.t. }\;{\dot{l}}_{s,\min}^{\prime }<{\dot{l}}_{s}^{\prime }<{\dot{l}}_{s,\max}^{\prime } \end{array}$$ (1)

where *g*_*p*,*ls*_ is the proportional gain and the maximum velocity that a linear screw actuator can travel in is limited to a small value $\left(\left|{\dot{l}}_{s,\min}^{\prime }|=|{\dot{l}}_{s,\max}^{\prime }\right|\right)$ . The motor cable spooling $l_{c}^{\prime }$ is modeled as a first order lag system, i.e.

$$T_{s}{\dot{l}}_{c}^{\prime }+l_{c}^{\prime }=g_{p,l_{c}}\left(l_{c}-l_{c}^{\prime }\right)$$ (2)

where *T*_*s*_ is the settling time and $g_{p,l_{c}}$ is the proportional gain. In addition to the specified models and constraints, there are also limits placed on the position values of the actuator joints in order to set hard constraints defined by geometric limits.

### 3.2 End-effector dynamics

**FIGURE 4:**
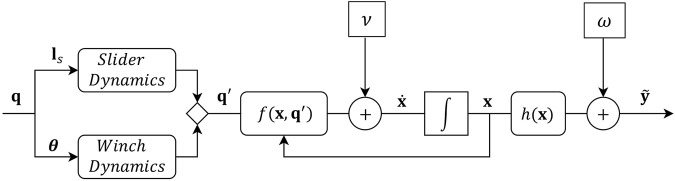
Plant model.

The cable failure is modeled as a linear drop in the cable stiffness of the failed joint, i.e., *k*_*i*_ → 0 over a small but finite time interval Δ*t*_*f*_. Thus if there is no change in winch angle as the cable fails, the tension in the corresponding cable falls. The full dynamic model of the rCDPR can be derived via the Lagrangian approach as

$$M{\ddot{x}}_{e}+D\left({\dot{x}}_{e}\right){\dot{x}}_{e}=J_{w}\left(x_{e},l_{s}^{\prime }\right)\;K_{q}\;\left[l_{p}\left(x_{e},l_{s}^{\prime }\right)-l_{c}^{\prime }\right]$$ (3)

where end-effector pose is given by $x_{e}={\left[\begin{array}{ccc} x & y & \phi \end{array}\right]}^{T}$ and $J_{w}\left(x_{e},l_{s}^{\prime }\right)$ is the pulling map or wrench Jacobian. The prismatic length, **l**_**p**_, comes from inverse kinematics. Both are dependent on the current pose, **x**_**e**_, and the realized geometric attachment points, $l_{s}^{\prime }$ , while the joint state stiffness matrix, *K*_*q*_, is a diagonal matrix with the elements (*k*_1_, *…* , *k*_4_). *M* is the mass/inertia matrix whose coefficients are informed by the mass and shape of the end effector. This formulation ignores the effects of Coriolis forces. The damping coefficients of the damping matrix *D* are minor and expected to be a result of surface friction alone. An effect of low cable stiffness and damping constants is that oscillations induced due to system dynamics in operation or during cable failure do not dissipate easily. A feedforward controller for such a system would be intractable. The complete dynamic system is shown in Figure 4.

#### 3.2.1 First order motion model

The states of the end-effector are given by $x={\left[\begin{array}{cc} x_{e} & {\dot{x}}_{e} \end{array}\right]}^{T}$ . The resulting non-linear system model is given by

$$\begin{array}{ll} \dot{x}\left(t\right) & =f\left(x\left(t\right),q^{\prime }\left(t\right),t\right)+\omega \\ \tilde{y} & =h\left(x\left(t\right),t\right)+v \end{array}$$ (4)

where ***ω*** and **v** are zero mean Gaussian vector processes accounting for model and measurement delta correlated noise. The non-linear vector functions *f* and *h* emerge as

$$\begin{array}{ll} f\left(x,q^{\prime }\right) & =\left[\begin{array}{c} {\dot{x}}_{e}\\ M^{-1}J_{w}\left(x_{e},l_{s}^{\prime }\right)K_{q}\left(l_{p}\left(x_{e},l_{s}^{\prime }\right)-l_{c}^{\prime }\right)-M^{-1}D{\dot{x}}_{e} \end{array}\right]\\ h\left(\text{x}\right) & =\left[\begin{array}{c} x_{e} \end{array}\right] \end{array}$$ (5)

where the explicit dependence on time has been dropped for ease of notation. The measurement model assumes that unbiased noisy position measurements are directly available, for instance by processing and filtering information from an on-board IMU or a range-based localization system.

### 3.3 Model assumptions for estimation and control

The motion models employed in the Extended Kalman Filters for the IMM (Section. 3.2) and the LQR (Section 5.4) use Eq. 4 and 5 as the motion model. Three motion models are employed to describe the seven working modes 4.1. For each working mode, the effect of the corresponding cable failure is incorporated in the dynamic model through the loss of stiffness in the respective cables. However, the model does not consider the true stiffness constant value from the plant model but instead employs static parameters corresponding to the different failure modes. In addition, it does not consider the model for actuator dynamics, but instead accepts a measurement of the current joint state, ${\tilde{q}}^{\prime }=q^{\prime }+v^{\prime }$ , where **v′** is modeled as a zero mean Gaussian delta correlated noise. On the other hand, the LQR assumes that the joint input to the motion model is the desired joint state. This is further discussed in Section 5.4.

The kinematic feedforward controller does not use the dynamic models and instead relies on the computation of the forward and inverse kinetostatic solutions for the redundant system. The cable stiffness model used to compute the desired cable tensions is approximated to consider the true cable length instead of the free cable length $\left(k=\frac{k_{0}}{l_{p}}\right)$ . This was done to remove the direct dependence of the cable stiffness parameter on *l*_*c*_ which is a control variable.

These assumptions add unmodeled uncertainties to the control and estimation problem. The complexity introduced by this bears resemblance to similar challenges associated with real-world experimental implementations. In addition, only a single measurement source (at the end-effector) is considered. It must be emphasized that the simulated measurement noise is unbiased and Gaussian. The deteriorating effect of measurement bias (such as IMU drift) on most estimation approaches is well known and well explained and are not considered in this work. Sensors such as ultrasonic sensors, GPS devices or visual systems can help eliminate bias. The selected measurement noise characteristics in this work was not based on simulations of a real measurement device.

## 4 Estimation of failure states

### 4.1 Failure mode classification

**FIGURE 5:**
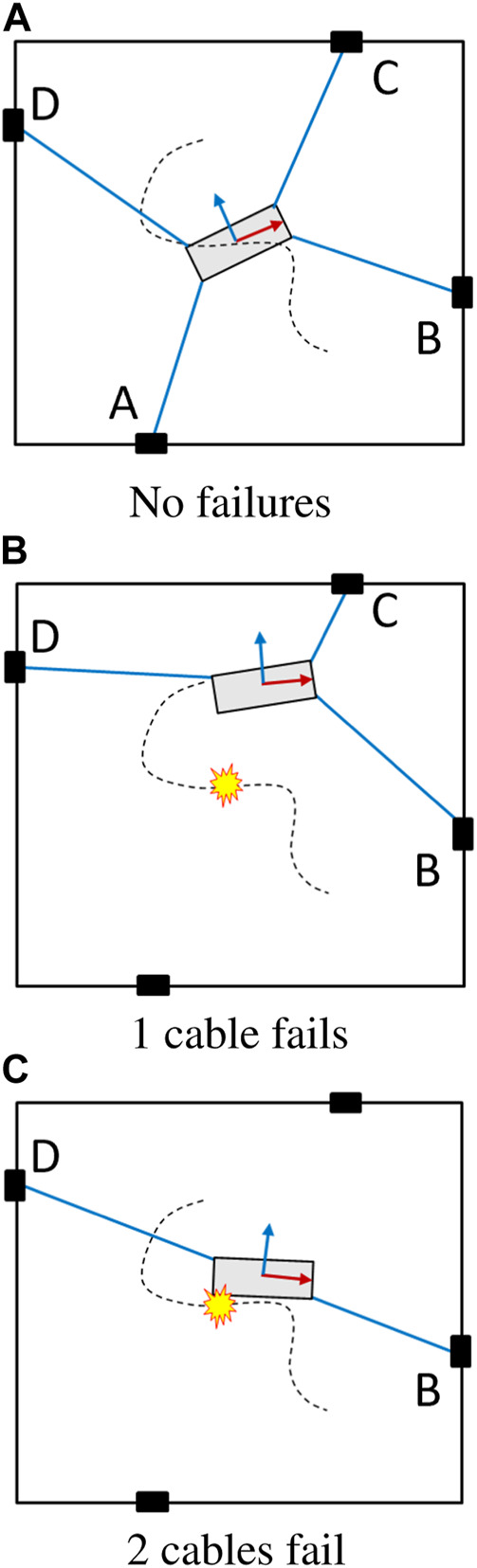
The 3 kinds of failures in a 4-PRPR CDPR **(A)** No failures **(B)** 1 cable fails **(C)** 2 cables fail.

Cable failure in a CDPR reduces the degrees of freedom (DOFs) or the quality of the DOF (i.e. manipulability or dexterity) available at the end-effector. We control only 2 DOFs at the end-effector for the four-cable planar CDPR model in this paper. The total working modes considered can be described by 3 motion models (i) an over-constrained CDPR with 4 cables, (ii) a fully constrained CDPR with 3 cables and (iii) and under-constrained CDPR with two cables (Figure 5).

Therefore, there are 7 total working modes (mode 1) no cables have failed, (modes 2–5) only cable A, B, C, or D fail, (mode 6) cables A and B have failed, (mode 7) cables C and D have failed. If any other failure combination occurs, significant areas of the workspace become unreachable and are considered to be complete failure modes (i.e. cannot be recovered from). If the sliders were not constrained to geometric limits and free to move like mobile robots or drones, then these additional failure modes can also be considered.

### 4.2 Interactive multiple model estimation

**FIGURE 6:**
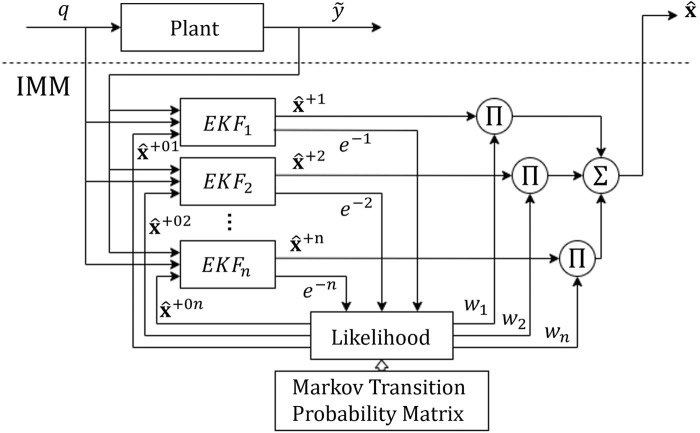
The Interactive Multiple Model (IMM) filter.

An interactive multiple model filter (IMM) is a dynamic estimator (Blom and Bar-Shalom (1988)) which can be used when model changes appear suddenly or gradually over time, providing means for failure detection. The process flow of an IMM is shown in Figure 6.

In general, the bank of filters in an IMM can be realized by a variety of estimation approaches, i.e., Kalman Filters (KF), Extended Kalman Filters (EKF), Unscented Kalman Filters (UKF), Particle Filters (PF), et cetera. In this study, we use an EKF formulation in which the error dynamics are approximated by a first order Taylor series expansion about the estimated state, $\hat{x}$ , i.e.

$$F=\frac{\partial f\left(x,u\right)}{\partial x},\;H=\frac{\partial h\left(x\right)}{\partial x}$$ (6)

Here, *F*_6 × 6_ is determined numerically by a complex step derivative approximation and *H*_3 × 6_ is simlpy given by $\left[\begin{array}{cc} I_{3\times 3} & 0_{3\times 3} \end{array}\right]$ , since we directly measure the end-effector’s position. In this study, the EKF is applied in the discrete time domain; hence, the Euler-Maruyama discretization of the motion model yields.

$$x_{k}=\underset{\phi \left(x_{k-1},q_{k-1}^{\prime }\right)}{\underbrace{x_{k-1}+f\left(x_{k-1},q_{k-1}^{\prime }\right)\Delta t}}+\omega_{k}\sqrt{\Delta t}$$ (7)

$${\tilde{y}}_{k}=h\left(x_{k}\right)+v_{k}$$ (8)

where $\tilde{y}\in R^{m}$ is the measurement vector, $\omega_{k}∼N\left(0,Q_{k}\right)$ denotes the process noise with covariance $Q_{k}\in R^{n\times n}$ , and $v_{k}∼N\left(0,R_{k}\right)$ is the measurement noise with covariance $R_{k}\in R^{m\times m}$ . The sampling time is denoted by Δ*t*, and *k* is the current time step.

Let M be the number of all working modes in a CDPR at the *k*th time step. Then, the input to the filter is the current measurement and mixed state estimate ${\hat{x}}^{+0}$ . The algorithm broadly consists of four stages (Gill (2019)).

1. *Interaction and Mixing:* The weights and the estimates from the last cycle are mixed as per their associated Markov transition probabilities given by the Markov transition matrix, *A* (also sometimes called the Markov switching matrix). The predicted probability for the filter to end up in mode *j* in each cycle, given that it was in the mode *i* during the previous cycle is given by

$$\begin{array}{ll} w_{k}^{\left(i|j\right)} & =\frac{1}{{\bar{c}}_{k}^{j}}w_{k}^{\left(i\right)}a_{ij}\\ {\bar{c}}_{k}^{j} & =\overset{M}{\underset{i=1}{\sum }}w_{k}^{\left(i\right)}a_{ij} \end{array}$$ (9)

with ${\bar{c}}_{k}^{j}$ being the normalization factor, and where *a*_*ij*_ is the Markov transition probability from mode *i* to mode *j*. As the true transition probabilities are unknown, the matrix *A* is a symmetric positive definite matrix of scalars that are tuned experimentally. The selected values have a significant impact on the response time and sensitivity in responding to cable failures. High values at the diagonals may cause the IMM to be sluggish whereas evenly distributed values along the whole matrix may cause the system to be overly sensitive to change. The mixed initial state estimate at the start of the current time step is provided by

$$\begin{array}{ll} {\hat{x}}_{k}^{+0j} & =\overset{M}{\underset{i=1}{\sum }}w_{k}^{\left(i|j\right)}{\hat{x}}_{k}^{+i}\\ P_{k}^{+0j} & =\overset{M}{\underset{i=1}{\sum }}w_{k}^{\left(i|j\right)}\left[\left({\hat{x}}_{k}^{+i}-{\hat{x}}_{k}^{+0j}\right)\right.\\ & \;\;\;\;\;\left.{\left({\hat{x}}_{k}^{+i}-{\hat{x}}_{k}^{+0j}\right)}^{T}+P_{k}^{+i}\right] \end{array}$$ (10)

2. *Model Specific Filtering:* The mixed initial estimates are fed into the EKF and processed in two steps, i.e.

• Propagation:

$$\begin{array}{ll} {\hat{x}}_{k}^{-j} & ={\hat{x}}_{k}^{+0j}+f\left({\hat{x}}_{k}^{+0j},q_{k}^{\prime }\right)\Delta t\\ P_{k}^{-j} & =\Phi_{k}^{j}P_{k}^{+0j}\Phi_{k}^{jT}+Q_{k}\\ \Phi^{j} & =I+F_{k}^{j}\Delta t \end{array}$$ (11)

• And update:

$$\begin{array}{ll} K_{k}^{\left(j\right)} & =P_{k}^{-j}H_{k}^{jT}{\left[H_{k}^{j}P_{k}^{-j}H_{k}^{jT}+R_{k}\right]}^{-1}\\ {\hat{x}}_{k}^{+j} & ={\hat{x}}_{k}^{-j}+K_{k}\left[{\tilde{y}}_{k}-h\left({\hat{x}}_{k}^{-j}\right)\right]\\ P_{k}^{+j} & =\left[I-K_{k}H_{k}\right]P_{k}^{-j} \end{array}$$ (12)

Here, ‘+’ denotes the *a posteriori* estimate whereas ‘−’ denotes the *a priori* quantity before the update. The likelihood of a measurement is then given by

$$\begin{array}{ll} p\left({\tilde{y}}_{k}|{\hat{x}}_{k}^{-j}\right) & =\frac{1}{{\left[\det\left(2\pi E_{k}^{-j}\right)\right]}^{1/2}}\\ & \;\;\cdot \exp\left[-\frac{1}{2}e_{k}^{-jT}{\left(E_{k}^{-j}\right)}^{-1}e_{k}^{-j}\right]\\ \text{with}\;E_{k}^{-j} & =H_{k}^{j}P_{k}^{-j}H_{k}^{jT}+R_{k}\\ e_{k}^{-j} & ={\tilde{y}}_{k}-{\hat{y}}_{k}^{-j} \end{array}$$ (13)

and where $e_{k}^{-j}$ is the estimation error.

3. *Model Probability Update:* Now, the model probabilities are updated via the likelihood with subsequent normalization, i.e.

$$\begin{array}{ll} w_{k}^{j} & =w_{k-1}^{j}p\left({\tilde{y}}_{k}|{\hat{x}}_{k}^{-j}\right)\\ w_{k}^{j} & \leftarrow \frac{w_{k}^{j}}{\sum_{i=1}^{M}w_{k}^{j}} \end{array}$$ (14)

4. *Combination:* Finally, the updated estimate is given by

$$\begin{array}{ll} {\hat{x}}_{k}^{+} & =\overset{M}{\underset{j=1}{\sum }}w_{k}^{j}{\hat{x}}_{k}^{+j}\\ P_{k}^{+} & =\overset{M}{\underset{j=1}{\sum }}w_{k}^{j}\left[\left({\hat{x}}_{k}^{+j}-{\hat{x}}_{k}^{+}\right){\left({\hat{x}}_{k}^{+j}-{\hat{x}}_{k}^{+}\right)}^{T}+P_{k}^{+j}\right] \end{array}$$ (15)

The weight vector, **w**_*k*_, displays the importance associated with each model in the bank. For example, if there are no cable failures, the IMM will determine the first model (mode 1) to have the largest weight. This vector also informs the balance of joint inputs to the controller as described in the next section.

## 5 Control framework

The objectives of the control framework are to not only stably manipulate the end-effector for trajectory tracking, but to do so with constant velocity and reliability of trajectory recovery upon cable failure. An earlier effort Raman et al. (2022) deployed a feedforward PD based control law for task space trajectory error minimization, but this was considered insufficient for addressing some finer requirements. For example, in 3D printing tasks the end-effector is required to (i) return to the point of failure before continuing, (ii) maintain constant velocity during trajectory tracking and, (iii) minimize dynamic oscillations at the end-effector after cable failure.

While designing a path following controller for a CDPR, the end-effector can generally be modelled as a holonomic robot. However, in most CDPRs, the ability to control the orientations of the end-effector is severely limited by geometric constraints. Reconfigurability can be an advantage here, but since our system model limits the motion of this attachment point to a straight line with finite dimensions, the orientations are only moderately more controllable than a traditional CDPR. Thus, we drop these from consideration for control and seek to only align the desired 2-D end-effector velocity vector with the tangent of the required trajectory.

In this section we present waypoint following algorithm integrated with a velocity level path following controller to recover the trajectory from the break-point (i.e. the precise location on the trajectory where the cable fails). This framework allows the recovery algorithm to be completely reliable even in the event of false positives by the estimator. In addition, this section also details the challenges of cascaded control and redundancy resolution in both the static space and task space while manipulating a kinematically- and actuation-redundant parallel manipulator.

### 5.1 Path following

We first discretize and parameterize the reference trajectory into a set of constant arc-length waypoints $W_{s}$ .

$$W_{s}=\left\{p_{i}=\left(x_{i},y_{i}\right)\;i\in 1\to {\text{N}}_{i}\right\}$$ (16)

where *N*_*i*_ is the total number of waypoints. The constant arc-length waypoints are numerically determined from the path equation. This re-parameterization ensures that regardless of the chosen trajectory equation the distance between any two waypoints is never too large. Waypoints function as an ordered set of markers without time parameterization that can be dropped from the waypoint map as the robot traverses within a threshold of it. The robot always moves toward the next waypoint in the map regardless of cable failure or any other unpredictable disturbance at the end-effector that may temporarily navigate the robot away from the desired trajectory.

Next, we use a constant-velocity carrot following controller to traverse straight line intersections between any given pair of waypoints.

**FIGURE 7:**
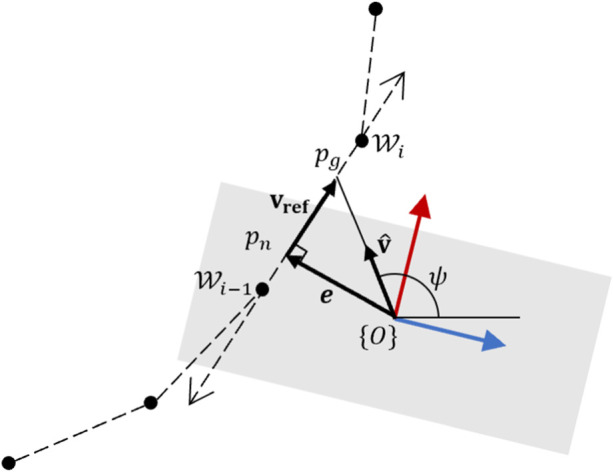
Constant velocity carrot-follower.

The shortest distance from the current position of the robot end-effector origin, shown in Figure 7 by frame {*O*}, intersects with the line segment at the point *p*_*n*_ and forms a normal vector **e** whose magnitude forms the cross-track error. If the projection of the robot frame origin {*O*} lies outside of the line segment, the shortest distance is simply the distance to the closest end-point. Therefore, the cross-track error vector is determined by first projecting the robot origin on the line segment **p** through a dot product and capping the magnitude of the projection $\|p\|\in \left[0,l_{W,i}\right]$ where $l_{W,i}$ is the length of the current line segment. If $o_{W}$ is the vector between frame {*O*} and $W_{i}$ , then the cross-track error vector is always given by $e=o_{W}+\|p\|\hat{p}$ . The reference velocity is a constant and is given by *v*_*ref*_. Thus the reference velocity vector which lies along the current line segment is $v_{ref}=v_{ref}\hat{p}$ . The desired direction of the velocity vector is given by **v**_**d**_ = **e** + **v**_*ref*_. The final desired velocity vector has its velocity always capped at *v*_*ref*_ and is therefore given by $v=v_{ref}{\hat{v}}_{d}$ .

As the cross-track error approaches zero, the desired velocity vector and the reference velocity vector are more closely aligned. The joint states required to achieve this velocity are computed through redundancy resolution-based inverse kinematics. When the end-effector reaches within a certain threshold of the next waypoint, the waypoint map $W_{s}$ updates by dropping the first value from the list. This strategy allows the robot to always move towards the second waypoint in $W_{s}$ , while the first waypoint serves as a marker for the connecting line segment to be tracked. If the robot snaps or moves off track, it still moves towards the second waypoint ensuring that the trajectory we want to track is traversed approximately from where it deviates. The choice of arc length chosen above determines the maximum size of the missed line segment.

### 5.2 Inverse kinematics and redundancy resolution

For a given end-effector pose, there are infinitely many solutions in the joint space of kinematically redundant CDPR. Considering that this system has two kinds of redundancies, actuation and kinematic redundancy, the solutions for determining the optimal joint states can be quite complex. In this study, a feedforward kinematic controller was applied and as a result, the redundancy resolution schemes and joint determination use kinematic models.

#### 5.2.1 Infeasibility avoidance

Singularity avoidance is an important part of the kinematic redundancy resolution control that determines the joint states. With CDPRs, it is additionally of importance to avoid infeasibilities in the wrench space. i.e., the configurations of the robot should always maintain positive cable tensions. Recent approaches use maximization of the volume of the wrench polytope Bosscher et al. (2006) (or the Available Wrench Set, AWS) as the cost function for ensuring this feasibility Rasheed (2019); Xiong et al. (2022). The available wrench polytope is a description of the wrench or manipulability ellipse that, unlike Yoshikawa’s measure of manipulability Yoshikawa (1985), provides a more accurate and scaled representation of the true wrench space. This is especially useful for CDPRs, where negative joint forces cannot be realized. However, the calculation of the AWS is computationally expensive and can still result in solutions that settle within local minima. For this reason, we choose to look at the traditional manipulability and wrench feasibility indices together by selecting the pareto-optimal solution maximizing both.

Yoshikawa’s measure of manipulability quantifies the twist and wrench capabilities of the end-effector. In 3 dimensions, the manipulability for translational and rotational motion is considered independently, and this holds true for 2 dimensions as well. Since we do not wish to control the yaw motion of the end-effector, we only consider the eigenvalues *σ* ∈ {*σ*_*x*_, *σ*_*y*_} of the twist Jacobian ${J_{w}}^{T}\left(x_{e},l_{s}\right)$ corresponding to *x* and *y*. The measure of manipulability is then given by

$$\lambda =\frac{\sigma_{\min}}{\sigma_{\max}},\;0\leq \lambda \leq 1$$ (17)

For an *n*-DOF CDPR with *m* cables, the wrench quality index *κ* indicates the quality of wrench feasibility of the system. For a fully constrained cable robot it is simply the ratio of the smallest and largest tensions required to maintain a pose Pott and Bruckmann (2013). For a redundantly constrained CDPR, it is the largest value that this ratio can take. In simple terms, the more evenly distributed the tensions in the CDPR are to maintain the pose, the better its wrench quality index will be. This index is calculated as follows.

• Let *J*_*w*_ be the wrench Jacobian for an *n* − *DOF* CDPR with the dimensions *n* × *m* where the number of cables, *m* ≥ *n* + 2. Most poses in this workspace can be achieved with only *n* + 1 cables, so many feasible solutions of tensions may involve setting tensions in *m* − *n* − 1 cables to zero and lead to a wrench quality index of zero. However, a better tension set can be determined for the same pose.
• Assume a matrix $J_{i}$ whose columns are a subset of the columns of *J*_*w*_ such that $J_{i}$ is *J*_*w*_ with a set of *m* − *n* − 1 columns dropped. Thus for *m* columns in *J*_*w*_ there will be ^*m*^ *C*_*n*+1_ combinations of $J_{i}$ .
• Let $n_{i}=\text{null}\left(J_{i}\right)$ . If every element in *n*_*i*_ has the same sign, then this vector contains the minimum set of cables that must be tensioned in order to maintain a pose (the tension in the remaining cables is zero). Expand the vector *n*_*i*_ such that zeros are inserted into the vector for every dropped column. For example, if *n* = 2 and *m* = 4, and $J_{1}$ is a reduced matrix of *J*_*w*_ with the first column dropped. Then $n_{1}={\left[\begin{array}{cccc} 0 & n_{1,1} & n_{1,2} & n_{1,3} \end{array}\right]}^{T}$

• Assemble all such vectors *n*_*i*_ whose elements contain the same sign and eliminate the negative signs *n*_*i*_ = −*n*_*i*_ iff *n*_*i*,*j*_ < 0 *∀ j*.
• Sum all the assembled *n*_*i*_ that meet these criteria into a single vector **z** of the dimensions *m* × 1.
• Drop all the elements in **z** that are zero. If the number of remaining elements are less than *n* + 1 then the wrench quality index, *κ* = 0. If *κ* is 0, then the system is not wrench feasible.
• If the number of elements is greater than or equal to *n* + 1, then

$$\kappa =\frac{\min\left(\left|z\right|\right)}{\max\left(\left|z\right|\right)}$$ (18)

For a fully constrained CDPR, the null space of the wrench Jacobian in one dimensional, so Eq. 18 is directly applied on the null vector (if the elements all contain the same sign. Otherwise *κ* = 0).

Cable failure can cause a sudden change in the instantaneous static workspace of the robot. The following waypoint on the desired trajectory can often lie outside of this new workspace. The sliders form the kinematically redundant joints that drive the robot away from singularities where necessary, but sometimes if the desired velocities at the end-effector are large enough or at an unusual angle, the sliders may be unable to move fast enough to compensate and the controller inadvertently pushes the robot into a singularity. To prevent this, we constrain the magnitude of the maximum allowable velocity with dependency on *κ*,

$$v=v_{\max}\;\sqrt{\kappa }\;\hat{v}$$ (19)

where $\hat{v}$ is the unit velocity direction vector. This is only utilized when the robot kinematics have placed the robot away from the trajectory, such as during the initialization. In normal operation for trajectory tracking the tracking velocities are slow enough that this is not a major concern.

#### 5.2.2 Redundancy resolution

This redundancy resolution scheme is applied on kinematic equations to determine an optimal position level joint solution, $q^{*}=\left[\begin{array}{cc} l_{s}^{*} & l_{c}^{*} \end{array}\right]$ . These inputs may not be dynamically feasible, nor is the feedback being actively measured, so they are instead locally thresholded to a velocity-limited value $q_{0}=\left[\begin{array}{cc} l_{s,0} & l_{c,0} \end{array}\right]$ . The optimal joint slider positions $l_{s}^{*}$ are determined from an objective function that seeks to maximize the manipulability and wrench quality at the end-effector. This aids in avoiding singularities as the sliders travel. The joint sliders are velocity-limited through a velocity thresholding function (Eq. 20). For the *i*th model (subscript *i* is dropped),

$$\begin{array}{ll} l_{s}^{*}\; & =\underset{l_{s}}{\max}\left(\kappa ,\lambda \right)\\ l_{s,0}^{k+1} & =l_{s,0}^{k}+\Delta l_{s,0}\\ \Delta l_{s,0}\; & =\min\left(\max\left(l_{s}^{*}-l_{s,0}^{k},v_{s,\max}\Delta t\right),v_{s,\min}\Delta t\right) \end{array}$$ (20)

where *κ* and *λ* are the wrench quality index and manipulability measure respectively. The slider velocity is given by *v*_*s*_. Feasible slider positions are required to calculate optimal cable winch positions for a given time-step. Ideally, slider positions and cable winching should be controlled in alternate timesteps in order to utilize slider measurements to calculate the desired cable winch position. However, by thresholding the slider velocities to |*v*_*s*, *min*_| = |*v*_*s*, *max*_| such that the control actions *l*_*s*,0_ lie within the linear region of the slider dynamics allows us to approximate control actions for sliders and cable winches within the same time-step. Any inaccuracies can be subsumed within model disturbances. The LQR feedback controller allows for enough leeway to compensate for these inaccuracies.

For the final motion model, the DOF at the end-effector reduces to one and the manipulability ellipse reduces to a line, making singularity avoidance a non-issue. Wrench feasibility in a single dimension is also trivially maintained. For this case, the objective function for the cables manipulates the end-effector along the single axis in the body frame, while the slider position depends on minimizing the error in a skewed axis (which is parallel to the direction of slider motion in the robot frame). Even for CDPRs with higher DOFs, *l*_*s*_ can be made to depend on a multi-objective function that simultaneously manipulates previously-uncontrollable DOFs while maximizing workspace and pose quality.

Cable failure can sometimes lead to slack in the remaining cables. While this property is not explicitly modeled in the system kinematics, its destabilizing effect can clearly be identified if the true winch position *l*_*c*_ is larger than the true cable length. This can be prevented by setting a minimum pretension ***τ***_*min*_ and utilizing the static redundancy to extract the desired tension in the cables. Given the desired end-effector position and $l_{s,0}^{k+1}$ , we determine the minimum positive tensions required to maintain this pose.

$$\tau^{*}=\min_{\tau }\tau^{T}\tau ,∋J_{w}\left(x_{e}^{k+1},l_{s,0}^{k+1}\right)\tau =0$$ (21)

The optimal winch positions, $l_{c}^{*}$ , are then determined in a straightforward fashion by considering the cable stiffness model,

$$l_{c}^{*,k+1}=\frac{K_{q}l_{p}^{k+1}}{\left(\tau^{*}+K_{q}\right)}$$ (22)

where **l**_*p*_ is the prismatic length or true (steady-state) length of the cables, determined from inverse kinematics. The joint angle velocities are not thresholded, so $l_{c,0}=l_{c}^{*}$ .

### 5.3 Forward kinematics

Given the desired joint state **q**_0,*n*_ of the *n*th working mode, the desired steady state end-effector pose **x**_0,*n*_ can be determined through forward kinematics which is implemented by minimizing the potential energy of the system.

$$x_{0,n}=\arg\min_{x_{0,n}}U=\frac{1}{2}{\left(l_{p}-l_{c_{0,n}}\right)}^{T}K_{q}\left(l_{p}-l_{c_{0,n}}\right)$$ (23)

$$\text{s.t.}\;\;\;l_{p}-l_{c_{0,n}}>0$$ (24)

where **l**_**p**_ is true cable length calculated for each value of **x**_0,*n*_. The cable tensions given by $K_{q}\left(l_{p}-l_{c_{0,n}}\right)$ must always be positive. If this condition is not met, it indicates that the initial joint state provided was not feasible which is generally a result of one or more cables being slack. Fortunately, in the feedforward control setting considered in this work, the desired winch position $l_{c_{0,n}}$ employed in the calculation of the forward kinematics is always less than the prismatic cable length **l**_**p**_. This calculation is not influenced by the true input **l**_**c**_ that is sent to the plant. Thus, although cable slackness can exist in the plant model, it is not encountered during the calculation of the forward kinematics.

The task recovery algorithm has a parallel bank of trajectory tracking controllers, each corresponding to a working mode. Each working mode, *n*, runs an independent kinematic controller that propagates the desired joint inputs in time such that it is the only working mode. The controllers use simple kinematic models for the different redundant CDPR motion models.

**FIGURE 8:**
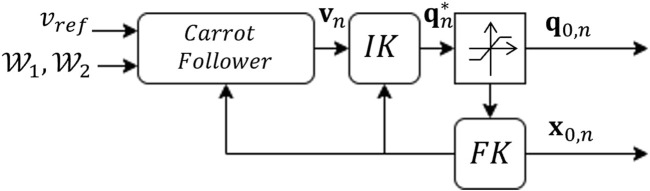
Feed-forward kinematic controller.

The control loop accepts the current joint inputs, **q** to the plant and determines the steady-state robot pose through forward kinematics for the *n*th model (Figure 8). The way point tracking algorithm accepts the current state estimate, $\hat{x}$ to update the waypoint and reference trajectory information if necessary. Through the carrot following algorithm, the desired task space state is determined and the redundancy resolution scheme is applied to determine the desired joint state, **q**_0,*n*_. This is propagated through forward kinematics again to determine the steady-state end-effector pose, **x**_0,*n*_.

### 5.4 LQR stabilization

Although the full system is highly non-linear, local stabilization (vibration dampening) is performed utilizing a Linear Quadratic Regulator. The LQR assumes the system model in Eq. 5 and ignores the actuator dynamics. This does not cause unwelcome destabilizing effects as the update frequency of the LQR is low compared to the dynamic joint controllers. The system model is linearized around the desired steady-state operating point (**x**_0,*n*_, **q**_0,*n*_) that results from a combination of forward and inverse kinematics in the feedforward controller described in Section 5.2. The local system dynamics then arise as

$$\begin{array}{ll} \delta x_{n,k+1} & =A_{n}\;\delta x_{n,k}+B_{n}\;\delta q_{n,k}\\ A_{n} & ={\left.\frac{\partial f_{n}\left(x,q\right)}{\partial x}\right|}_{\left(x_{0,n},q_{0,n}\right)},\;B_{n}={\left.\frac{\partial f_{n}\left(x,q\right)}{\partial q}\right|}_{\left(x_{0,n},q_{0,n}\right)} \end{array}$$ (25)

where *k* is the time-step index within the LQR feedback control loop. Please note that the desired steady-state operation point (**x**_0,*n*_, **q**_0,*n*_) is also time-varying, but at a much slower update rate than the LQR loop. The slow update rate of the feedforward controller that provides the desired steady-state operating point is useful here as the redundancy resolution algorithm is the most computationally expensive piece of the framework. Therefore, a zero-order hold assumption without loss of generality is implied in Eq. 25. The dynamic correction *δ* **q**_*n*,*k*_ for the kinematically optimal joint input **q**_*n*,*k*_ is now determined via a quadratic cost function, i.e.

$$\delta q_{n,k}=\arg\min_{q_{n}}\overset{\infty }{\underset{k=1}{\sum }}\delta x_{n,k}^{T}Q\delta x_{n,k}+\delta q_{n,k}^{T}R\delta q_{n,k}$$ (26)

subject to the dynamics in Eq. 25 and where Q and R are positive semi-definite and positive definite weighting matrices, respectively. The tuning of these terms depends on the desired overall impact of the corresponding states on the cost function as well as limits on the control effort. Here, the structure for Q and R has been chosen as diagonal. Furthermore, the diagonal terms for the joint inputs (R) are weighted equally. For Q , the entries corresponding to the velocities in *x* and *y* outweigh the other values, as it is desired to minimize velocity offsets dominantly. The resulting controller is then given by

$$q_{n,k}=q_{0,n}-K_{n}\delta {\hat{x}}_{n,k}=q_{0,n}-K_{n}\left({\hat{x}}_{k}-x_{0,n}\right)$$ (27)

where $K_{n}$ is the LQR gain resulting for the *n*th model while the estimated state ${\hat{x}}_{k}$ from the IMM framework has been employed as the feedback variable for the controller. When tuned well, the LQR stabilization not only mitigates dynamic disturbances due to the elasticity of the cables during normal operation, but also dampens significant oscillations from cable breakage quickly. An additional beneficial effect from the LQR inclusion is observed, as even slackness after failure is more efficiently eliminated. Here, the kinematic solution provides a new equilibrium point, and the LQR inherently rushes the system to arrive at this new stable kinematic solution, thus significantly aiding in maintaining positive cable tensions. For the over-constrained (4-cable) and fully constrained (3-cable) rCDPR models, only ***θ***_0,*n*_ is subject to LQR stabilization as this is enough to dampen perturbations in both *x*- and *y*-directions. For the under-constrained (2-cable) model, the redundant joints **l**_0,*n*_ are also utilized to dampen disturbance components orthogonal to the cable direction.

### 5.5 Input mixing

The bank of controllers independently determine the next steady-state input for each failure mode. The bank of LQRs generate optimal joint inputs that are mixed with the weighting matrix to the plant model. The final mixed input gives precedence to the dominant working mode identified by the IMM. Due to this mixing, the final mixed input from this stage will always be subject to some perturbation. However, this perturbation is considered a reasonable trade-off to accommodate a solution that is robust and reliable during cable failure conditions.

**FIGURE 9:**
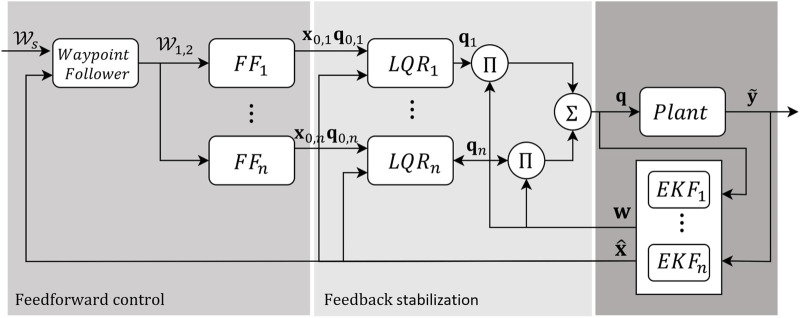
LQR stabilization and input mixing.

The mixed joint input is applied to the dynamic model (plant) and the measurements of the end-effector state inform the state estimation and weight vector through the IMM. The complete algorithmic framework is described in Figure 9.

## 6 Implementation and results

### 6.1 Simulation framework

The plant model described in Section 3 is perturbed with a process and measurement noise to mimic unmodeled dynamics. The cables are modeled as linear springs (where stiffness is a function of free cable length) with no mass and no sag, while all other model parameters are constants. Cable failure is modeled as a drop in cable stiffness to zero over a finite time. If the stiffness is zero, the cable can have no tension and thus has no impact on the platform dynamics. In addition, if the dynamic input to the system causes a situation where *l*_*c*_ > *l*_*p*_, the tension in the dynamic equation is set to zero to mimic cable sag. The plant dynamics and estimation run at a frequency of 1,000 Hz while the closed loop LQR feedback is run at 50 Hz. The feedforward kinematic model, which is the most computationally-intensive system, is run at 2 Hz. The entire framework is modeled in MATLAB. The model parameters are given in Table 1.

**TABLE 1:** Dynamic Model Parameters.

Parameters	Notation	Value	Units
End-effector dimensions	*b* × *h*	0.16 × 0.08	*m* × *m*
Base Span	*l*_*b*_	0.56	*m*
Mass	*m*	0.5	*kg*
Cable Stiffness Proportionality Constant	*k*_0_	5	*N*
Surface friction coefficient	*μ*	0.1	-
Gravitational constant	*g*	9.81	*m*/*s* ^2^
Minimum Cable Tension	*τ*_*i*_	0.5	*N*
Time period for cable failure	Δ*t*_*f*_	0.1	*s*
Process noise covariance	*Q*	$\left[\begin{array}{cc} 10^{-10}I_{3\times 3} & 0_{3\times 3}\\ 0_{3\times 3} & 10^{-3}I_{3\times 3} \end{array}\right]$	{*m*, *rad*, *s*}
Measurement noise covariance	*R*	10^−5^ *I*_3×3_	{*m*, *rad*}

### 6.2 Task recovery

**FIGURE 10:**
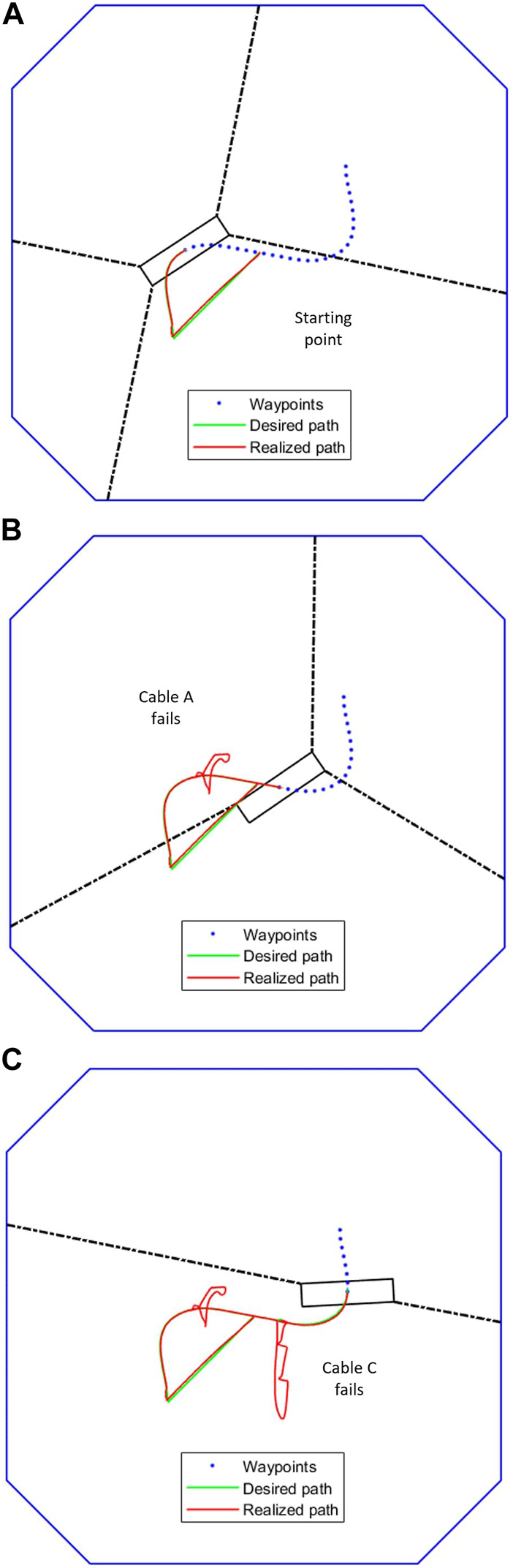
Trajectory tracking and recovery after cable failure **(A)** No failures yet **(B)** After 1 cable fails **(C)** After 2 cables fail.

Figure 10 demonstrates the application of the complete task recovery algorithm. The red trajectory shows the true path of the center of the end-effector while the green trajectory shows the desired path. The blue dots are the predefined waypoints. The failure of the first cable causes the end-effector to jump away from its current path, but the algorithm immediately responds by rearranging the remaining three winches and pulling the end-effector back. This breakaway from the trajectory and subsequent recovery with three cables is shown by the first kink in the red line part way through the trajectory in Figure 10B. The failure of the second cable and subsequent task recovery is seen further down the trajectory in Figure 10C.

**FIGURE 11:**
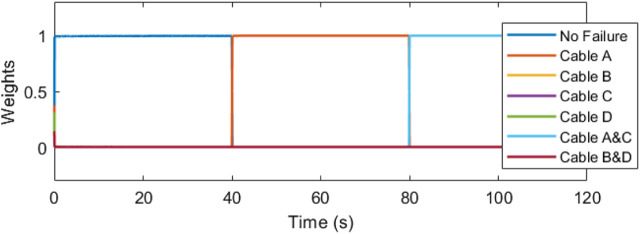
The weights denote the dominance of the corresponding cable failure case.

**FIGURE 12:**
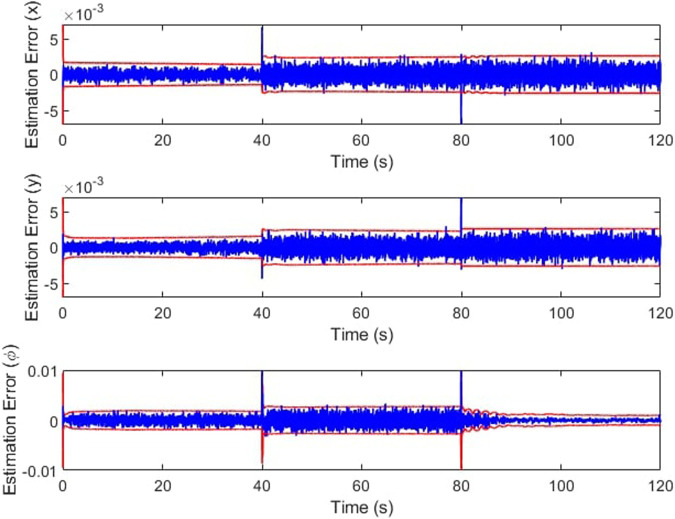
Estimation error over time.

The IMM correctly identifies the failure, mixing and switching the inputs as needed to automatically recover the trajectory tracking. In Figure 11, the weights vector correctly assigns the largest values to the current working mode. At the forty second mark, we can see that the weight vector has correctly identified the cable A failure, followed by the correct identification of the cable C failure at the 10 s mark. The estimation error plots are in Figure 12.

**FIGURE 13:**
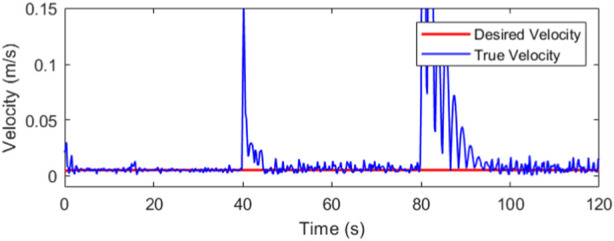
Desired and actual velocities at the end-effector.

The robot is maintained at constant velocity throughout its trajectory (Figure 13). The desired velocity during trajectory tracking is 0.005 m/s or 5 mm/s which is a reasonable speed for the dimensions of the workspace. During recovery, the end-effector velocity is not a constraint, so the controller, mainly through the efforts of the LQR, attempts to move the end-effector as fast as dynamically feasible.

**FIGURE 14:**
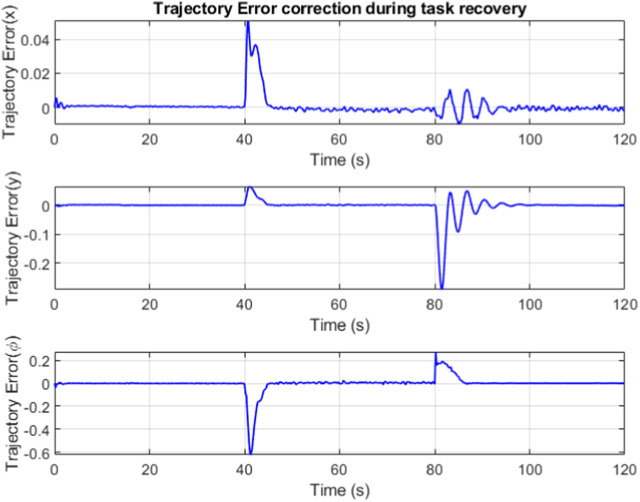
Trajectory error over time.

The trajectory error is minimized by the combined efforts of feedforward controller and the LQR. The banded regions in Figure 14 represent sections where the robot is in recovery mode, attempting to minimize the trajectory error while compensating for the changes in slider and winch position.

**FIGURE 15:**
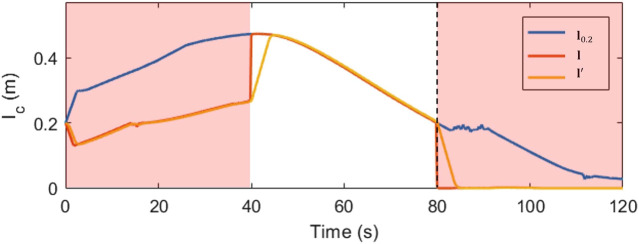
Desired, true and measured slider positions over time.

The robot does not enter singularities in the feedforward controller. Figure 15 shows the plots of a single slider position (slider C) for the second motion model. In the example trajectory shown in Figure 10 this model is weighted highest between the 40 s and 80 s timestamps. Thus the mixed output from the LQR, **l** begins to weight heavily in the favour of the control input **l**_0,2_ from model 2, while the true slider position **l**′ begins to catch up. When cable C breaks at the 80th second, this model begins to fall out of favor and the slider position deviates from this value.

**FIGURE 16:**
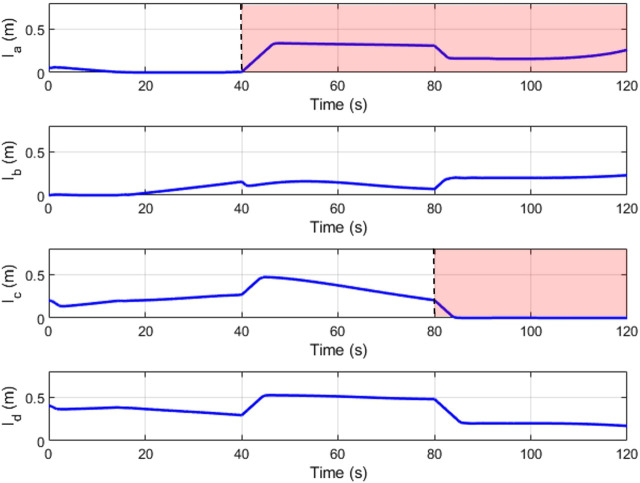
Slider positions (*l*_*s*_) over time.

**FIGURE 17:**
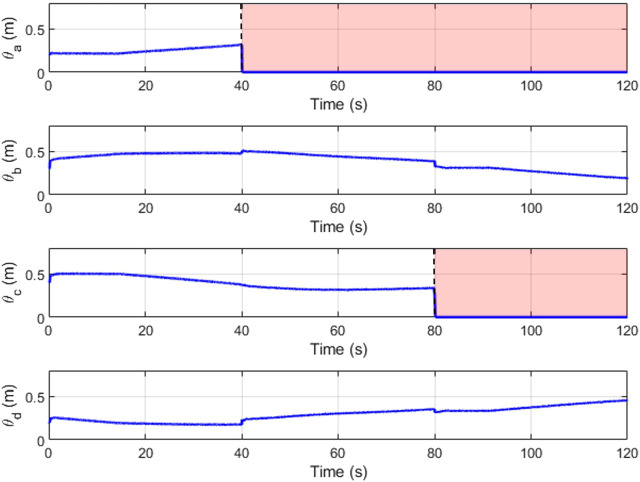
Winch positions (*l*_*c*_) over time.

**FIGURE 18:**
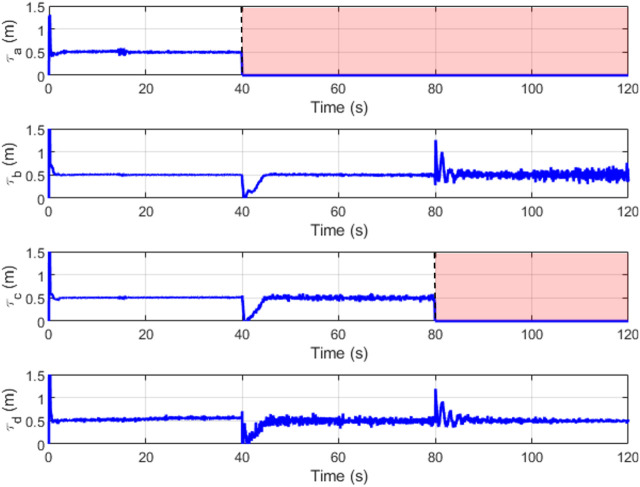
True cable tensions (*τ*) over time.

Figures 16, 17 depict the change in slider position and position angle of winch respectively. The shaded regions represent the failed state of the respective cable. The values of the slider and winch positions in these regions are perturbed by the combined attempts of the optimization to continue modulating this actuator and the weighted input mixing but has no effect on the end-effector as the corresponding cable is broken. This is seen clearly in the plots observing the true tension in the cables in Figure 18. The position values are not automatically settled to zero to account for cases where the cable failure may have been misidentified by the estimator.

A minimum value of pre-tension in the cables is maintained at 0.5 N as a good practice to eliminate the chances of encountering cable slackness. This tension is seen to be maintained throughout the run with the minimum required tensions in the remaining cables to propagate the end-effector along the trajectory. This demonstrates that the tension minimization scheme is working well. When a cable snaps, there are brief moments of cable slackness while the tensions re-distribute as the sliders reposition themselves and the winches wind within their designed dynamics. These dynamics do not affect the steady-state input from the feedforward controller and thus the LQR bears the burden of recovery.

**FIGURE 19:**
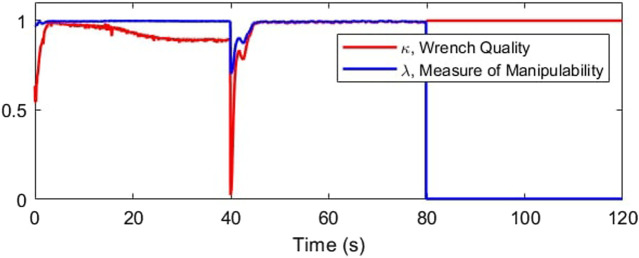
Maximizing workspace quality and manipulability.

The redundancy resolution optimization for the slider positions maximizes the manipulability and wrench quality. In Figure 19 we see these properties hold high values in the first two-thirds of the plot. These are the regions where the optimization-determined slider positions are actively applied. This optimization is no longer relevant for the two cable cases, where the slider position instead functions to replace the lost degree of freedom.

### 6.3 Comparison study

**FIGURE 20:**
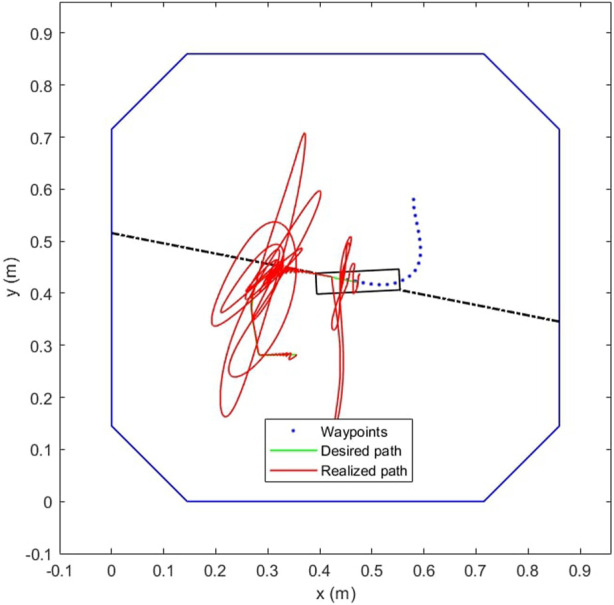
Trajectory tracking and recovery without LQR for oscillation damping.

For completeness, this section compares the results of the previous section with a feedforward controller applied directly on the system without the LQR. The update rate of the feedforward controller is 50 Hz and all other parameters are kept the same. The trajectory results are presented in Figure 20.

**FIGURE 21:**
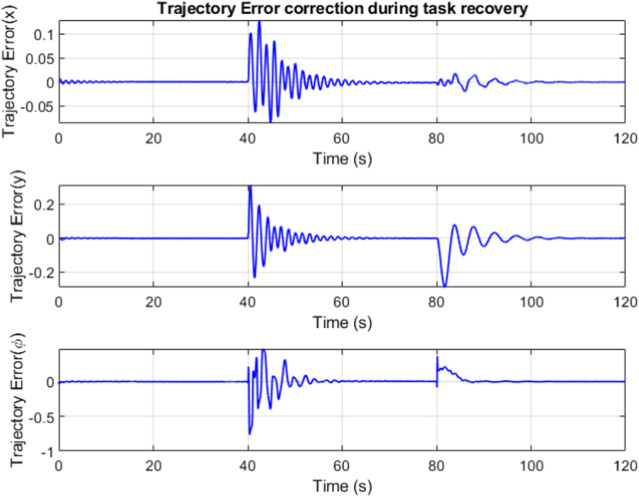
Trajectory error for the case without LQR for oscillation damping.

The trajectory error plot in Figure 21 indicates that the oscillations do eventually dampen and the kinematic controller is generally successful after this due to the slow velocities at the end-effector.

**FIGURE 22:**
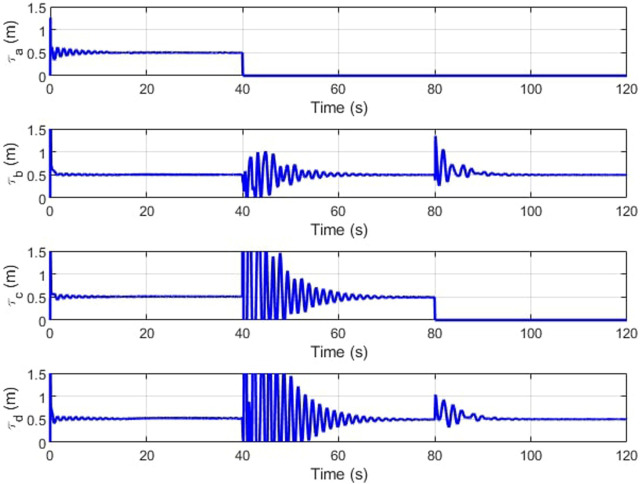
Cable tension for the case without LQR for oscillation damping.

The cable breakage causes the end-effector to swing causing periodic slackness in the cable tensions (Figure 22).

**FIGURE 23:**
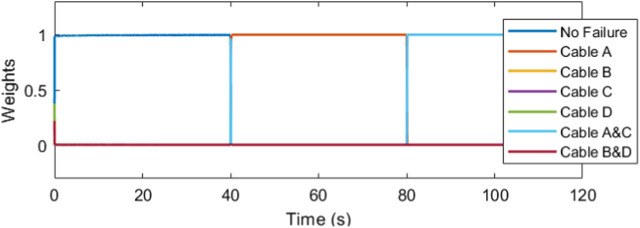
Model identification is unaffected.

The estimation depends solely on the end-effector measurements, so it is unaffected by the controller dynamics as seen in Figure 23.

## 7 Discussions

In this work we create an end-to-end control and estimation framework to simultaneously accomplish cable failure diagnosis and detection and cable failure tolerant control through the utilization of an interactive multi-model adaptive estimation algorithm and a bank of controllers to provide the mixed inputs. This framework is applied on a planar reconfigurable cable driven parallel robot (rCDPR); a CDPR with the ability to geometrically reconfigure its cable winch attachment points through the utilization of linear sliders. We are able to create a system that is (i) automatic: it does not require the faulty actuators to register a fault during the event of a cable failure; (ii) robust; although it requires only measurement data from the end-effector to estimate the correct failure state, this data can be quite noisy and the models utilized in the filters do not need to be perfect (iii) reliable; when the correct failure state is weighed higher, the corresponding inputs associated with this state have a higher impact on the inputs to the system which improves the overall estimate. However, if an incorrect state is weighed high, the corresponding inputs will create dynamics that do not match the expected system profile which allows the correct state to eventually be identified. In addition, the inclusion of the waypoint following algorithm ensures that the robot always reconnects with the trajectory at the approximate location where it deviated. The utilization of a feedforward controller over a dynamic feedback control helps improve the overall stability of the system. In addition, since the feedforward framework does not require the current state of the system, it can also be designed to be an offline controller that can help improve computation times and realize a real-time control system. The LQR feedback controller ensures not only that the robot dynamics due to elasticity during normal operation are minimized but also attempts to reconfigure the system as quickly as possible when a failure mode is estimated. Although the input mixing at this stage can cause some perturbation at the end-effector, this effect is considered a reasonable trade-off for the overall advantages of dynamic stabilization that the controller offers.

## Data Availability

The original contributions presented in the study are included in the article/Supplementary Material, further inquiries can be directed to the corresponding author.
